# Lichens Bite the Dust – A Bioweathering Scenario in the Atacama Desert

**DOI:** 10.1016/j.isci.2020.101647

**Published:** 2020-10-07

**Authors:** Patrick Jung, Karen Baumann, Dina Emrich, Armin Springer, Vincent J.M.N.L. Felde, Stefan Dultz, Christel Baum, Marcus Frank, Burkhard Büdel, Peter Leinweber

**Affiliations:** 1 Applied Logistics and Polymer Sciences, University of Applied Sciences Kaiserslautern, Carl-Schurz-Straße 10-16, 66953 Pirmasens, Germany; 2 Faculty of Agricultural and Environmental Science, Soil Science, University of Rostock, Justus-von-Liebig-Weg 6, 18051 Rostock, Germany; 3 University of Freiburg, Faculty of Environment and Natural Resources, Chair of Applied Vegetation Ecology, Tennenbacher Str. 4, 79106 Freiburg, Germany; 4 Medical Biology and Electron Microscopy Centre, University Medicine Rostock, Strempelstraße 14, 18057 Rostock, Germany; 5 Department Life, Light and Matter, University of Rostock, 18051 Rostock, Germany; 6 Department of Soil Science, Faculty of Organic Agricultural Sciences, University of Kassel, Nordbahnhofstr. 1a, 37213 Witzenhausen, Germany; 7 Institute of Soil Science, Leibniz Universität Hannover, Herrenhäuser Str. 2, 30419 Hannover, Germany; 8 Plant Ecology and Systematics, University of Kaiserslautern, Erwin-Schrödinger-Straße, 67663 Kaiserslautern, Germany

**Keywords:** Earth sciences, Geomicrobiology, Earth-Surface Processes, Weathering, Biogeoscience

## Abstract

Bioweathering mediated by microorganisms plays a significant role in biogeochemical cycles on global scales over geological timescales. Single processes induced by specific taxa have been described but could rarely be demonstrated for complex communities that dominate whole landscapes. The recently discovered *grit crust* of the coastal Atacama Desert, which is a transitional community between a cryptogamic ground cover and a rock-bound lithic assemblage, offers the unique chance to elucidate various bioweathering processes that occur simultaneously. Here, we present a bioweathering scenario of this biocenosis including processes such as penetration of the lithomatrix, microbial responses to wet-dry cycles, alkalinolysis, enzyme activity, and mineral re-localization. Frequently occurring fog, for example, led to a volume increase of microorganisms and the lithomatrix. This, together with pH shifts and dust accumulation, consequently results in biophysical breakdown and the formation of a *terrestrial protopedon*, an initial stage of pedogenesis fueled by the *grit crust*.

## Highlights

• A microbial community in the Atacama Desert mediates diverse bioweathering processes
• Lichens, algae, and heterotrophic microbes lead to the deterioration of small stones
• Detection of swelling actions, shifts in pH, enzyme activity, and mineral transports
• Formation of a terrestrial protopedon as initial stage of soil on landscape scale

## Graphical Abstract

**Figure:**
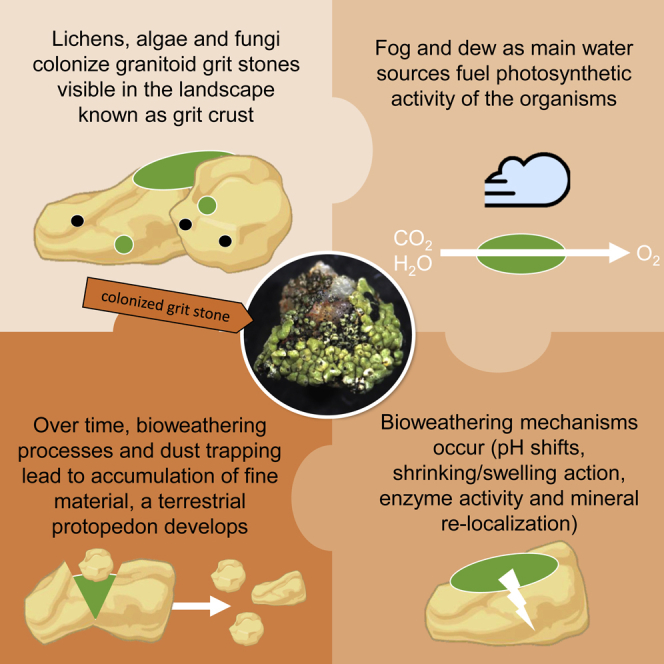

## Introduction

Early development of terrestrial ecosystems often includes interactions between cryptogamic lithobiontic communities (cyanobacteria, green algae, lichens, and fungi together with other heterotrophic organisms), the lithomatrix of rocks and the given climatic conditions (Mergelov et al., 2018; Mitchell et al., 2019). Since the occurrence of life on Earth, the interplay between the biotic and abiotic world is amongst the most ancient processes shaping the earth's surface. For example, the symbiotic interaction between algae and fungi in lichens probably dates back to the Precambrian 400 million years ago (Taylor et al., 1995) although there is evidence for a later origin of modern lichens dating back 320 million years (Lumbsch and Rikkinen, 2017; Nelsen et al., 2020). However, the terrestrial organic carbon (C_org_) pool of early soils has been fueled to a large extend by the biological activity of cyanobacteria, later supported by algae and fungi until land plants appeared roughly 300 million years ago.

Nowadays, lithobiontic communities are still well known from arid environments such as the Atacama Desert (Azúa-Bustos et al., 2011), where not only external surfaces of hard rocks but also cracks and fissures may be conquered by microorganisms using bioreceptive characteristics of the internal rock structure (Wierzchos et al., 2015). In this region, fog and dew are the main water sources for photosynthetic activity (e.g. Lehnert et al., 2018). Consequently, the alteration and transformation of the lithomatrix by metabolism and bioweathering processes of phototrophs can be expected (Weber et al., 2011). This was described for extreme habitats such as Antarctica or ancient Mayan buildings (Ortega-Morales et al., 2016) and similar interactions are very likely to occur in the Atacama Desert, but reports are still missing. Single processes of biogeophysical and biogeochemical weathering by (cyano-)bacteria, green algae, lichens, and fungi are well studied (reviewed in Salvadori and Municchia, 2016; Chen et al., 2000). This includes, e.g., the biological transformation of clay minerals, e.g. by K depletion of interlayers of mica/illite and oxidation of structural Fe(II) of less weathering resistant silicates such as biotite as well as the dissolution of phosphate salts such as apatite (Smith, 1978; Wierzchos and Ascaso, 1998; Chen et al., 2000). Many studies investigated the weathering of quartz, one of the most common minerals which is an oxide where the atoms are linked in a framework of SiO_4_ that makes quartz one of the most stable minerals in terms of weathering. Various microorganisms were found to mediate biochemical weathering processes of rock-forming minerals such as the excretion of pH shifting substances that interfere with the lithomatrix (acidolysis or alkalinolysis), the production of chelating compounds such as siderophores (complexolysis; Daghino et al., 2010) or the manipulation of the redox potential via extracellular enzymes (Włodarczyk et al., 2016). Although phototrophs are a focal point of interest for bioweathering processes more and more studies are showing a hidden potential in heterotrophic microorganisms such as basidiomycete fungi (Kirtzel et al., 2020) or bacteria (Matlakowska et al., 2012). However, so far, no study has shown bioweathering activity as the sum of these complex biochemical and biophysical weathering factors during the development of terrestrial ecosystems at the landscape scale, but rather single processes have been documented (e.g. Souza-Egipsy et al., 2004; Büdel et al., 2004).

Contrary to lithobiontic communities which colonize rock surfaces, biocrusts establish on and between unconsolidated inorganic and organic soil compounds. This means that the biocrust organisms grow at the surface of sediment and/or soil or within the upper few mm of the upper soil horizon, rather than directly on the more or less weathered parent rock (Belnap, 2003; Garcia-Pichel et al., 2016). However, investigations on weathering mechanisms and rates mediated by biocrust organisms are still in their infancy. This may be due to a greater predisposition of the lithic habitat to weathering and erosional processes, whereas the soil habitat is already a first result of these processes (Garcia-Pichel et al., 2016). Estimations on weathering rates are not only hindered due to erosional processes but also in cases where the original situation before biocrust formation cannot be reconstructed (a prerequisite for calculation of mass balances) due to the presence of different shares of parent rock materials, e.g. from Aeolian dust inputs from different petrological regions (local sources versus those from long-distance transport). One of the few described processes in the context of biocrust-mediated weathering is the leaching and re-localization of elements mobilized by biogenic processes in deeper soil horizons (Beraldi-Campesi et al., 2009) as well as the accumulation of organic matter, which triggers further pedogenetic processes such as the formation of organo-mineral associations (Dümig et al., 2014). Due to the fact that these are permanent processes in biocrusts, Beraldi-Campesi et al. (2009) speculated that allochthonous (Aeolian dust) inputs compensate for elemental loss in the upper soil mm to maintain the nutrition of biocrusts in the long run. Some rock outcrops can be highly resistant against biochemical weathering due to its coarse mineral grain size, low specific surface area, and the presence of less vulnerable minerals to (bio)chemical weathering such as quartz, alkali feldspars and muscovite in granitic rocks. If this is the case fine-sized deposited Aeolian dust particles containing high shares of layer silicates and also organic compounds can be a major phase for element mining and energy recovery by biocrust organisms.

Recently, a unique transitional community between lithobiontic and biocrust biocenoses, termed *grit crust*, growing around and inside granitoid grit stones of about 6 mm in size (locally called *maicillo*) that are paving the ground in broad areas of the coastal Atacama Desert, was described (Jung et al., 2020). The vivid colonization of the grits by mainly lichens causes a blackish pattern of several square meter large patches on the ground that can be seen across the landscape (hereafter called *black grit*) right beside less colonized grits forming patches that appear whitish (hereafter called *white crust*). The reasons for the scattered presence of the biocenosis in the landscape is still not clarified but seems to be more complex than the simple relation between topography, fog water deposition, and colonization rate (Jung et al., 2020). So far, this biocenosis has been detected to cover locally between 20 and 80% of the 350 km^2^ National Park Pan de Azúcar, highlighting its significance in terms of ecosystem services such as nutrient acquisition, water retention or erosion prevention (Jung et al., 2020).

The tight relation between the organisms and the lithomatrix of the grits enabled us to describe a bioweathering scenario mediated by lichens, cyanobacteria, green algae, and fungi that potentially leads to pedogenesis on landscape scales. We hypothesized that complex and various interactions between the *grit crust* organisms and deposited dust, the soil compounds and grit interfaces lead to a physical breakdown of the latter and an accumulation of fine soil particles. For these reasons, an interdisciplinary approach consisting of soil analyses such as mineral composition, dust composition, bioweathering assays such as long-term and short-term pH shifts induced by green algal lichen photobionts, enzyme activity and the shrinking-swelling action of colonized grit stones induced by water was applied. In particular we A) tested to which extend swelling and shrinking of the organisms induced by frequently occurring fog can lead to the physical breakdown of the grit stones, B) tested whether pH shifts caused by the organisms can in turn promote etching of substrate particles and C) characterized the texture and elemental content of the accumulated material which contributes to pedogenesis.

## Results

**Figure 1:**
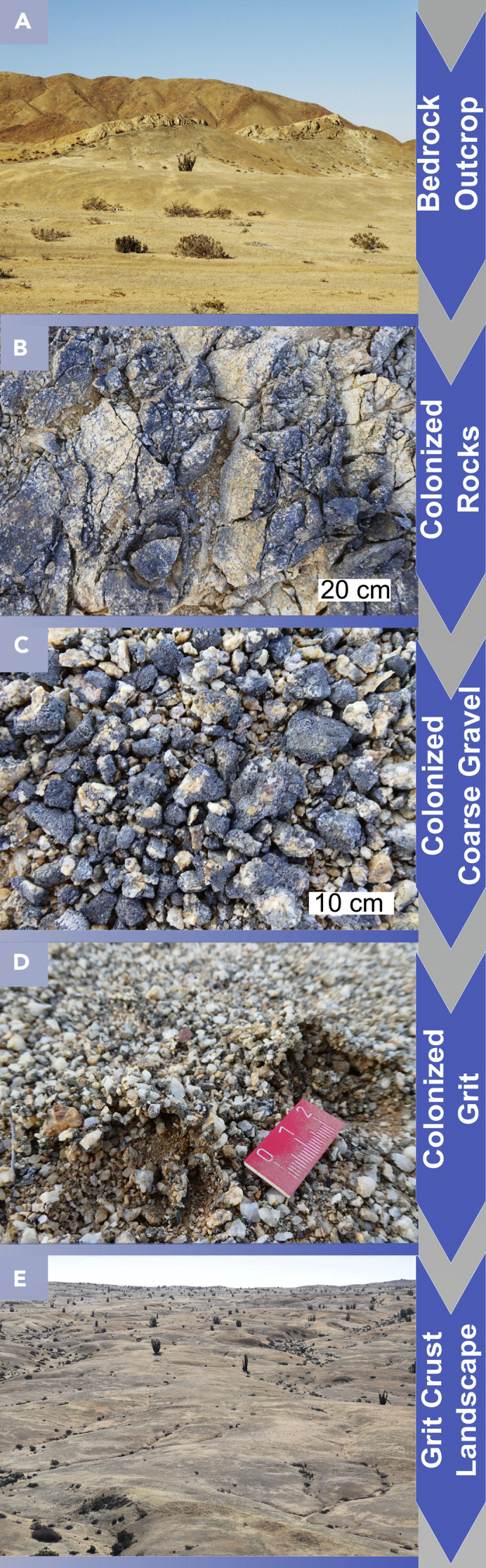
Chronosequence of Bioweathering in the Landscape of the National Park Pan de Azúcar, Atacama Desert (A) Bedrock outcrop made of granitoid material. (B) Colonized rocks in close vicinity of the bedrock outcrop. Scale bar indicates 20 cm. (C) Colonized coarse gravels in close vicinity of the rocks. Scale bar indicates 10 cm. (D) Grit stones concatenated by organisms. (E) Colonized grit causing blackish patterns in the landscape.

Based on field observations in the National Park Pan de Aúzcar that is situated in the coastal range of the Atacama Desert we found evidence which led to the following bioweathering chronosequence presented in Figure 1. This sequence depicts the idea of microorganisms such as lichens, algae, fungi, and other heterotrophic organisms colonizing boulders of parent rock in the landscape (Figures 1A and 1B). Subsequently the interplay between abiotic and biotic weathering processes led to the deterioration of the rocks into smaller fragments that were also colonized till the current snapshot in time resulting in the ‘grits’ (Figure 1C). The grits have a size of roughly 6 mm and cover large areas at least in the National Park Pan de Azúcar (Jung et al., 2020). They appear as blackish patterns on the ground in the landscape when they are colonized by microorganisms such as lichens. Our study investigated the bioweathering processes mediated by this association of microbial life and the grits, also called grit crust.

### Mineralogical Analyses of Black and White Grit Samples (First cm)

The electrical conductivity value observed for the white grit was 2.4-fold higher compared to the black grit (Table 1). Also, the pH-value of the white grit was higher (8.0) compared with that of the black grit (6.9) (Table 1). In the aqueous extracts of the samples, the most common anion was Cl^−^, and for the cations Na^+^ (Table 1). Comparing black and white grit samples, marked differences existed in the concentration of different anions and cations.

**Table 1:** Anion and Cation Concentration in the 1:10 Aqueous Extract, Electrical Conductivity (EC), and pH of Black and White Grit Samples

Sample	F^-^	Cl^-^	NO_2_^-^	Br^-^	NO_3_^-^	PO_4_^3-^	SO_4_^2-^	Ʃ Anions	Clay [%]
**[mmol**_**c**_**L**^**−1**^**]**									

Black grit	–	1.05	–	–	0.026	0.044	0.138	1.258	3.0
White grit	–	3.24	–	–	0.038	–	0.248	3.526	6.4
Sample	**N**a^**+**^	**NH**_**4**_^**+**^	**K**^**+**^	**Mg**^**2+**^	**Ca**^**2+**^	**Ʃ cations**	**EC [**μS cm^−1^]	**pH**	

**[mmol**_**c**_**L**^**−1**^**]**									

Black grit	0.774	0.030	0.265	0.091	0.166	1.326	188	6.9	
White grit	2.761	–	0.322	0.114	3.506	3.506	446	8.0	

Chemical analysis of the <2 and >2 μm fractions of black and white grits revealed highest SiO_2_ contents in the >2 μm fraction, where the primary mineral quartz was typically observed (Table 2). K_2_O was highest in the <2 μm fraction indicating that K was not only located in feldspars but also in illite. In this fraction, also the percentage of P_2_O_5_, Fe, Mn, Cu, and Zn was higher compared to the >2 μm fraction.

**Table 2:** Chemical Composition of the <2 μm and >2 μm Fraction

Sample	MgO	Al_2_O_3_	SiO_2_	P_2_O_5_	K_2_O [%]	CaO	Ti	Fe	Mn	Cu	Zn
Black grit <2 μm	0.54	10.12	47.19	0.074	3.35	0.30	0.149	2.80	0.03	0.022	0.030
Black grit >2 μm	0.78	6.81	68.28	0.025	2.32	0.27	0.082	0.90	0.01	0.003	0.002
White grit <2 μm	0.92	7.32	55.08	0.060	2.52	0.23	0.098	2.20	0.04	0.013	0.019
White grit >2 μm	0.78	4.64	77.51	0.029	1.69	0.23	0.058	0.74	0.02	0.003	0.002

**Figure 2:**
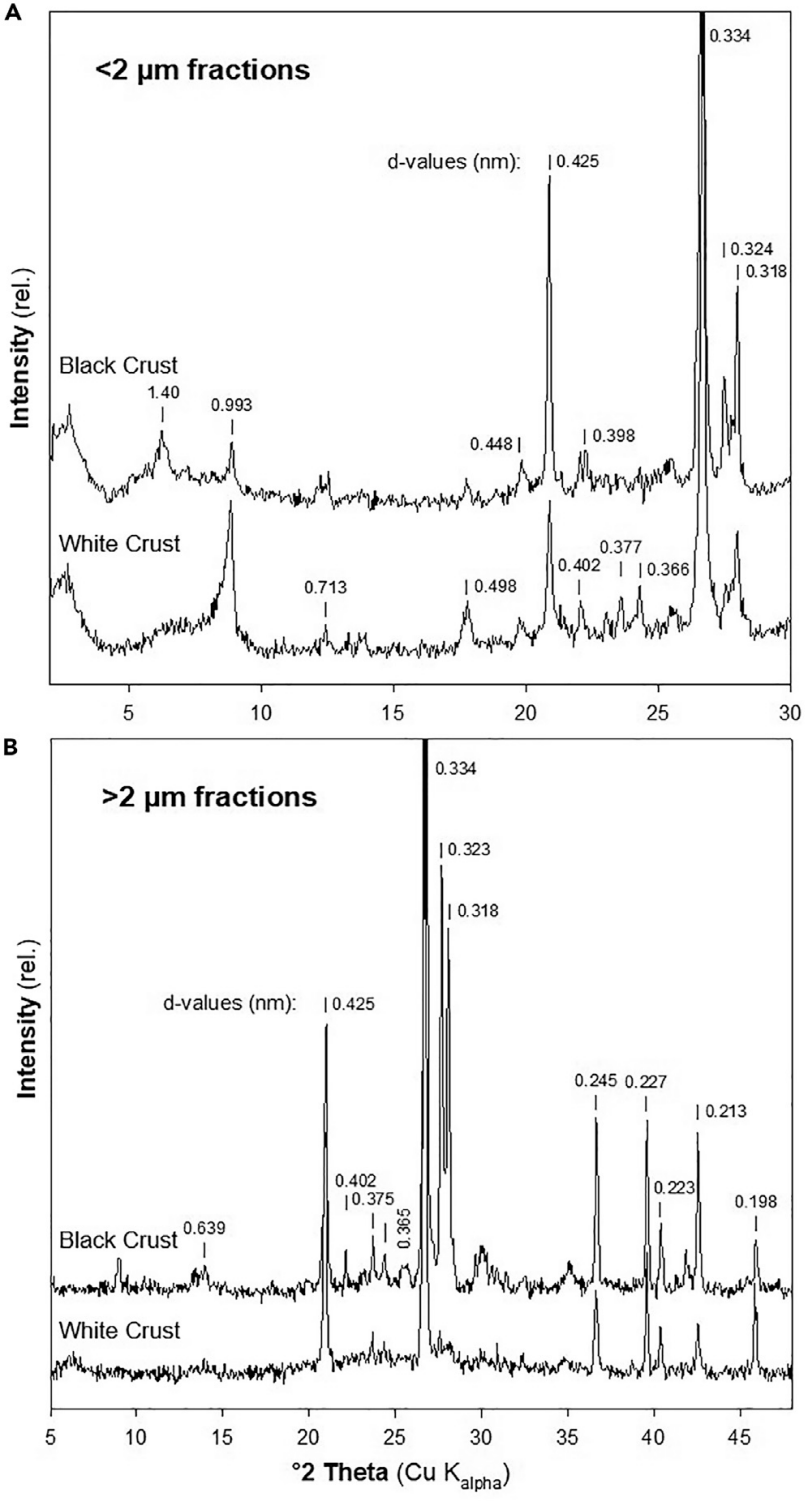
X-ray Diffraction Patterns of the Grit Soil Samples (A) This represents the <2 μm fraction and (B) the >2 μm fraction of black and white grit samples. Mg-saturated samples were used for the <2 μm fraction.

For black and white grit samples, diffraction patterns for the <2 μm fraction revealed the presence of illite (peak maximum at 0.99 nm) (Figure 2). In the black grit sample a 2:1 layer silicate phase was indicated by the diffraction peak at 1.40 nm. Traces of kaolinite were indicated by small interferences at 0.713 nm. The presence of relatively high amounts of quartz could be deduced from strong interferences at 0.425 and 0.334 nm. Peak maxima at 0.323 and 0.318 nm could be assigned to feldspars and were most marked in the <2 and >2 μm fraction of the black grit sample. The two clay minerals, typical for arid soils, palygorskite with a strong X-ray diffraction peak at 1.04 to 1.05 nm and sepiolite with a strong maximum at 1.24 nm, were not identified in the samples. Carbonates were absent as well. In the >2 μm samples, the presence of quartz was indicated in addition to the interferences at 0.425 and 0.334 nm by maxima at 0.245 and 0.227 nm. From the SiO_2_ content in the >2 μm fractions it was derived that quartz had higher shares in the white grit than black grit sample (77.5 and 68.3% SiO_2_, respectively).

### Texture and Elemental Composition of the First and Second cm of Substrate and of Dust

**Figure 3:**
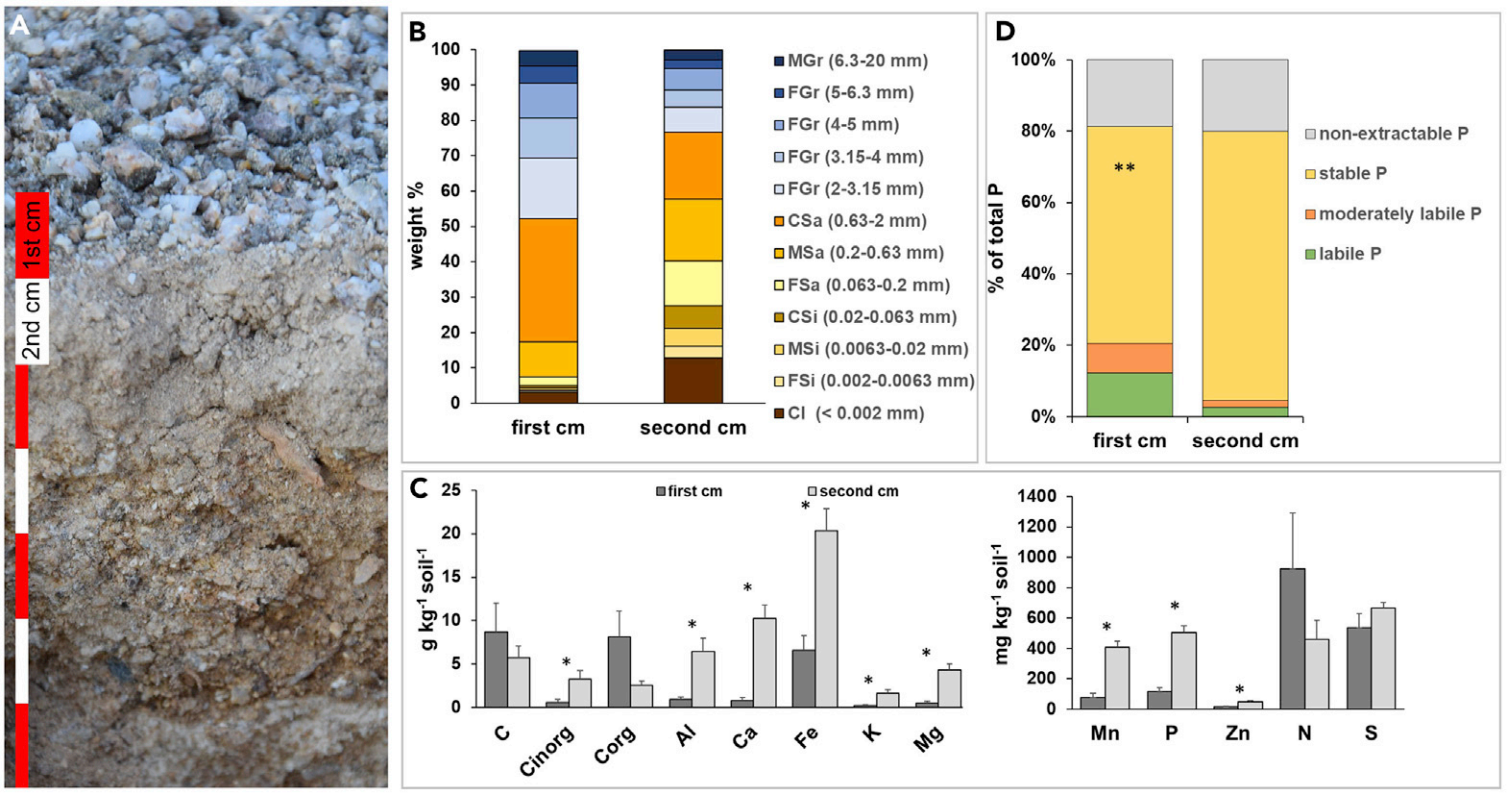
Texture and Elemental Composition of the First and Second Centimeter of a Grit Crust Profile (A) Upper centimeters of a *terrestrial protopedon* profile with accumulated fine material between and underneath the grit stones; red and white scale bars represent 1 cm each; (B) Texture of the first and second centimeter of the profile; C = coarse, M = middle, F = fine, Gr = gravel, Sa = sand, Si = silt, Cl = clay; (C) Elemental composition of the first and second centimeter of the profile represented as mean +/− standard deviation; asterisks indicate significant differences between an elemental concentration of the first and second centimeter, *p* ≤ 0.1, paired t test; (D) P pools as percentage of total P in the first and second centimeter of the profile; asterisks indicate significant differences between first and second centimeter within one fraction at ∗∗*p* ≤ 0.05, paired t test.

The texture of the first and second cm of the analyzed substrate (Figure 3A) was characterized by a high proportion of coarse material (50% > 2 mm), which included the colonized grit stones in the first cm of the profile (Figure 3B). The second cm of the profile mainly consisted of fine material (80% < 2 mm) including 12% of clay-sized particles.

Element contents as gained from inductively coupled plasma – optical emission spectroscopy after acid digestion were normally distributed at *P* ≤ 0.05, except for zinc (Zn) which only was normally distributed at *P* ≤ 0.1. The total elemental contents of Al, Ca, Fe, Mg, Mn, P, Zn, and C_inorg_ were significantly higher in the second cm compared to the first cm (Figure 3C). The increase in the second cm ranged between 2-fold (Al, Fe) to 7-fold (Ca). The total P content was 3-fold higher in the second cm compared with the first cm. Total C and C_org_, as well as total N and S were similar in the first and second cm.

Sequential P-fractionation showed that stable P was the dominant P-fraction in both depths. In the second centimeter, its proportion was significantly higher than in the first cm (Figure 3D). Labile and moderately labile P were by trend higher in the first cm.

### Fluorescence Microscopy of Colonized Grit Stones

**Figure 4:**
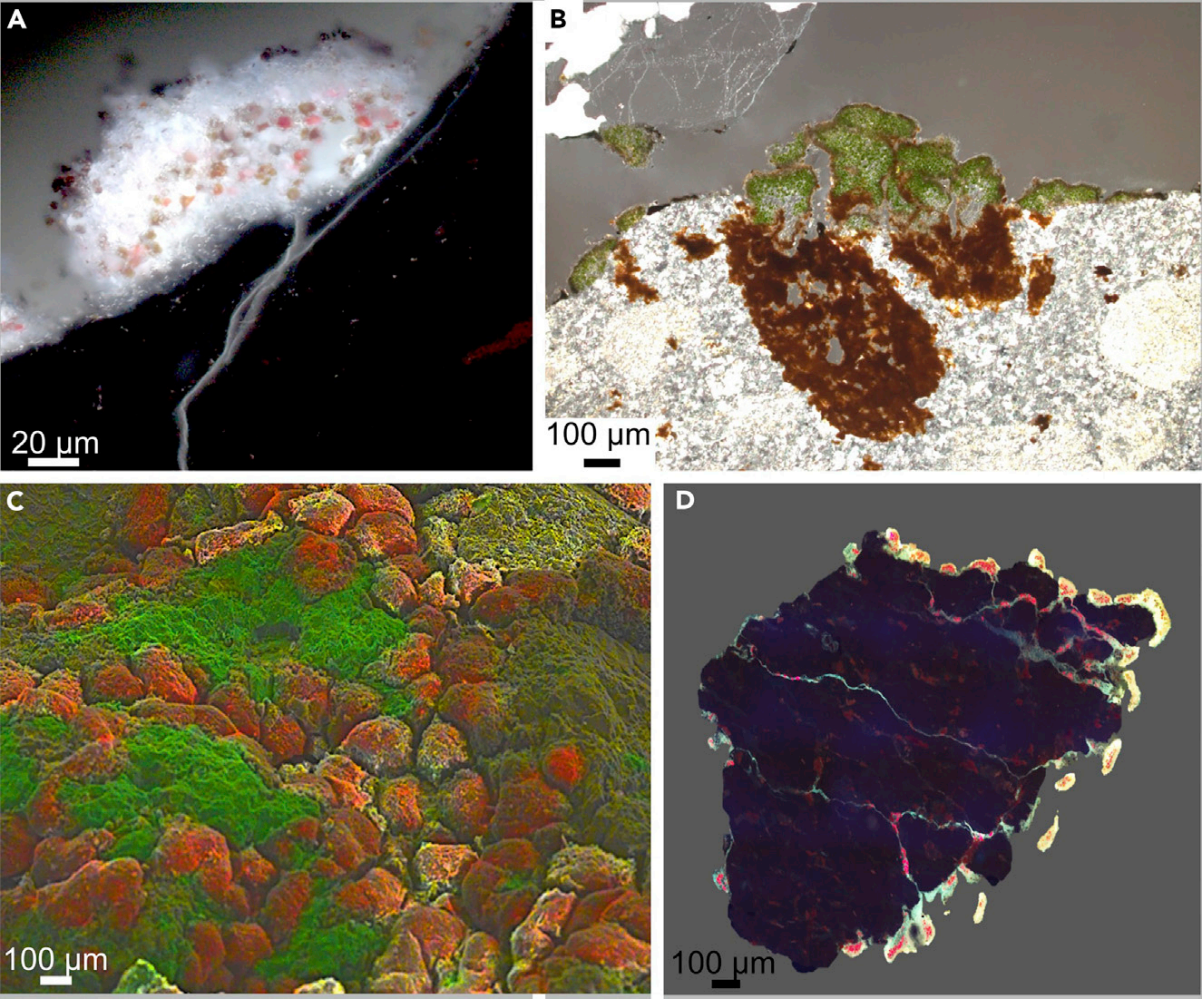
Evidence for Penetration of the Lithomatrix by Lichens (A) Fluorescence microscopy of grit stone showing fungal hyphae (white; chitin autofluorescence) penetrating the grit surface and green algal photobionts (red; chlorophyll autofluorescence) indicating the microstructures that potentially lead to a deterioration of the grit stones; (B) Light microscopy of grit cross section with lichen thalli (green) and cavities filled with brown particles underneath that demonstrate that lichens accumulate fine material; (C) Artificially colored SEM image with lichen thalli (red) embedded into the grit (green) showing that the lichen thalli are embedded into the surface of the grits; (D) Fluorescence microscopy of whole grit stone showing fungal hyphae (white; chitin autofluorescence) and green algal photobionts (red; chlorophyll fluorescence) inside of a grit stone. SEM, scanning electron microscopy.

Fluorescence microscopy showed black, white and red patterns with black color as the lithomatrix, white color as fluorescence of the fungus due to chitin in the cell walls and red color as chlorophyll fluorescence of the green algae (Figures 4A and 4D). Autofluorescence of green algae would not be visible from dead cells or remnants because chlorophyll rapidly degrades after cell death. Figure 4A demonstrates that lichens grew attached to the surface of the grit stones with single hyphae penetrating the grit. This phenomenon was frequently observed. In addition, most tunnels of single grits were found to be colonized by fungal hyphae and their green algal photobionts even reached the middle of the grits (Figure 4D). Light microscopy of grit thin sections indicated lichen thalli with their green algal photobionts growing tightly attached to the surface of the grit stones. Brown colored cavities beneath the lichens imply clay or Fe-oxides accumulation (Figure 4B). Scanning electron microscopy (SEM) images indicated that the lichen thalli (artificially colored in red) were embedded into the grit surface (artificially colored in green) (Figure 4C).

### X-Ray Microtomography

**Figure 5:**
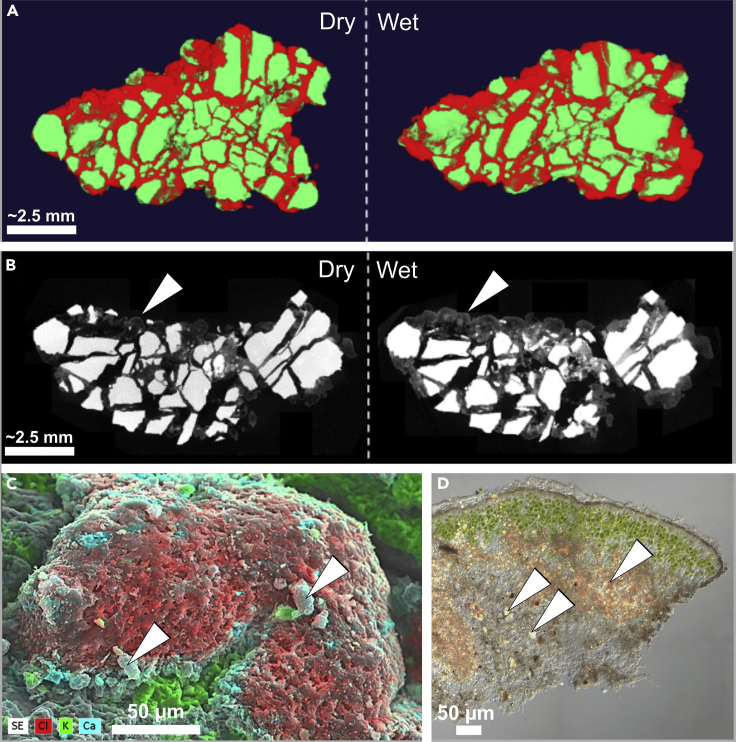
Effects of Fog and Dew Induced Wet-Dry Cycles and Mineral Accumulation on Colonized Black Grit (A) Schematic output of the segmented 3D model for the grit before (left) and after wetting (right). While the stones (green) did not change their volume, the lichen tissue (red) showed a considerable increase in volume, which is especially obvious for the inner regions between the stone fragments. (B) Gray scale tomograms of the grit before (left) and after wetting (right). Arrows indicate regions where swelling of the lichen tissue was most pronounced. (C) Artificially colored SEM-EDX image showing the presence of salts attached to a lichen thallus (arrowheads); (D) Microscopic cross section of lichen thallus showing incorporated minerals (arrowheads) as weathered residues. SEM, scanning electron microscopy; EDX, energy-dispersive X-ray spectroscopy.

Comparative X-ray microcomputer tomography (μCT) scans of a colonized grit stone in an air-dry status and after 12 hr of hydration revealed a change of lichen structure after wetting. Table 3 shows that the volume of the whole grit (stone including lichen) increased about 21% (Figures 5A and 5B). The volume of the lichen tissue alone, raised up to 55% of its initial value. The relative share of the lichen for the total grit volume increased from 38% in the dry state to 49% when the lichen was hydrated. The fact that the calculated volume of the stone enlarged only very slightly (by 0.13%) is an indicator for the reliability of the μCT data.

**Table 3:** Results of the Microcomputed Tomography Analysis

	Dry		Wet		% change	
	Volume [μL]	Surface area [mm^2^]	Volume [μL]	Surface area [mm^2^]	Volume	Surface area
Stone	41.21	453.70	41.26	461.52	±0.13	±1.73
Lichen	25.75	653.31	39.99	756.82	±55.29	±15.84
Whole grit	66.96	429.75	81.26	534.66	±21.35	±24.41

Volume and surface area of the colonized grit components (stone and lichen) and whole grit before and after wetting. Note that the surface area of the whole grit is only referring to the outer surfaces.

The surface area of the whole grit enlarged by 24% (Table 3). This increase is mostly caused by the enlargement of the lichen surface, as the stone surface did expand only by about 2%, which corresponds to the very small increase in the stone's volume. Further, most of the surface expansion happened at the outer surface of the grit. The internal surface area can be calculated when subtracting the surface area of the whole grit from the sum of the surface of mineral phase and lichen, which also have internal surfaces. Interestingly, while the whole grit surface expanded by 24%, the internal surface stayed almost the same with an increase of less than 1% (dry: 677.27 mm^2^; wet: 683.69 mm^2^).

### SEM-energy-dispersive X-ray spectroscopy and Element Composition Analyses

**Figure 6:**
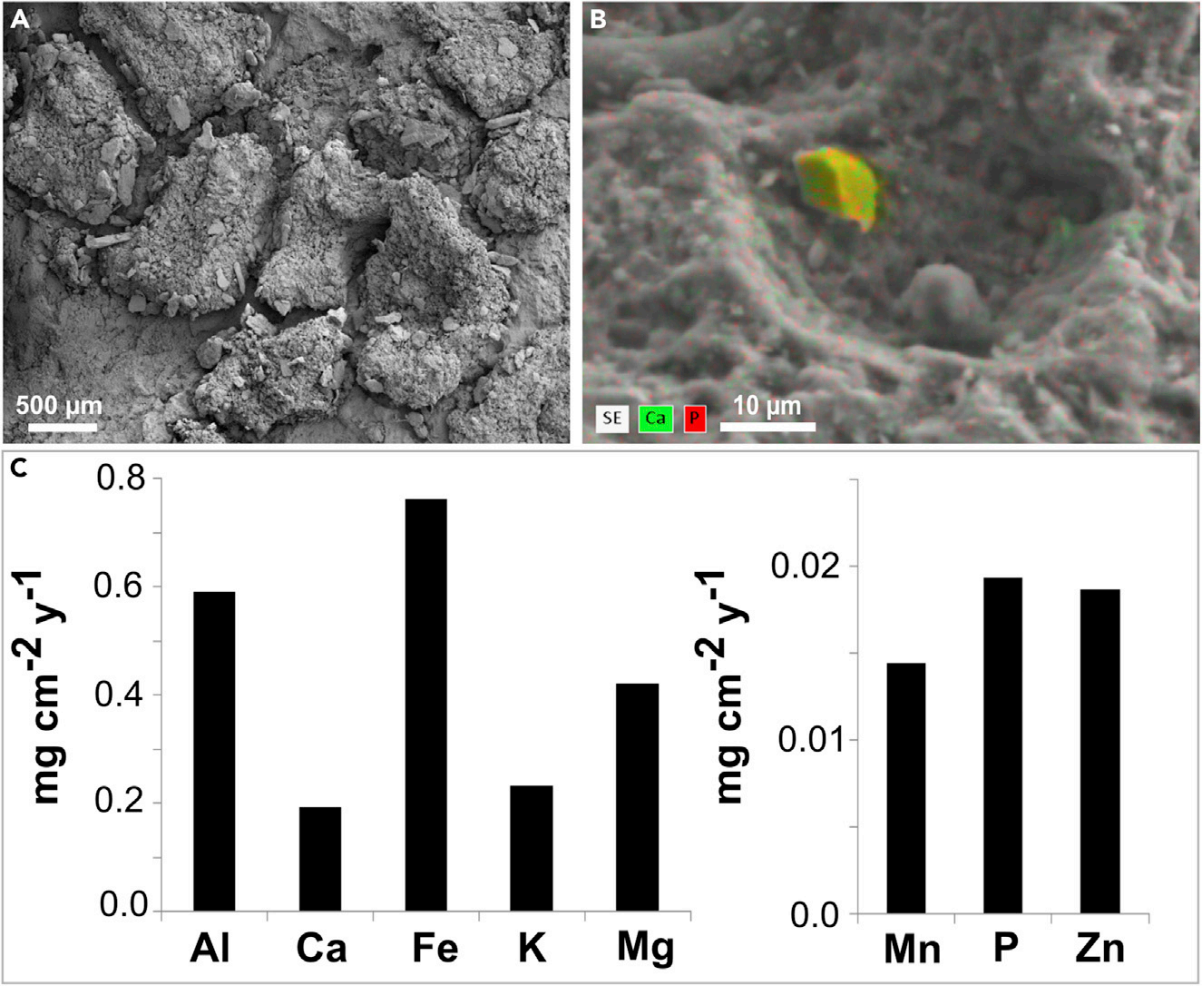
Mineral Accumulation on Grit Surface and Elemental Dust Composition (A) SEM image of lichen thalli attached to grit with mineral particles from dust on the cortex of the thalli; (B) Artificially colored close-up SEM-EDX image of dust particle on top of lichen thalli showing a grain consisting of Ca and P compounds; (C) Elemental deposition by dust. SEM, scanning electron microscopy; EDX, energy-dispersive X-ray spectroscopy.

SEM with energy-dispersive X-ray spectroscopy (SEM-EDX) indicated high abundance of Cl and Ca at the external surface of the lichen thalli while mainly potassium K was found at the surface of the grit (Figure 5C). Light microscopy of a thin section of detached lichen thalli revealed high amounts of incorporated mineral particles e.g. iron oxides (brownish colored) within the medulla of the thallus (whitish colored) underneath the photobiont layer (greenish colored) (Figure 5D). Further, SEM images showed mineral particles of varying sizes embedded into the cortex and on top of the lichen thalli (Figure 6A). The elemental composition of these particles included high abundances of Ca and P as revealed by SEM-EDX (Figure 6B). Dust contained mainly Fe and Al but also 0.019 mg P cm^−2^ y^−1^ (Figure 6C).

### Long-term pH Measurements of Photobiont Cultures and Enzyme Activity of Colonizing Organisms

**Figure 7:**
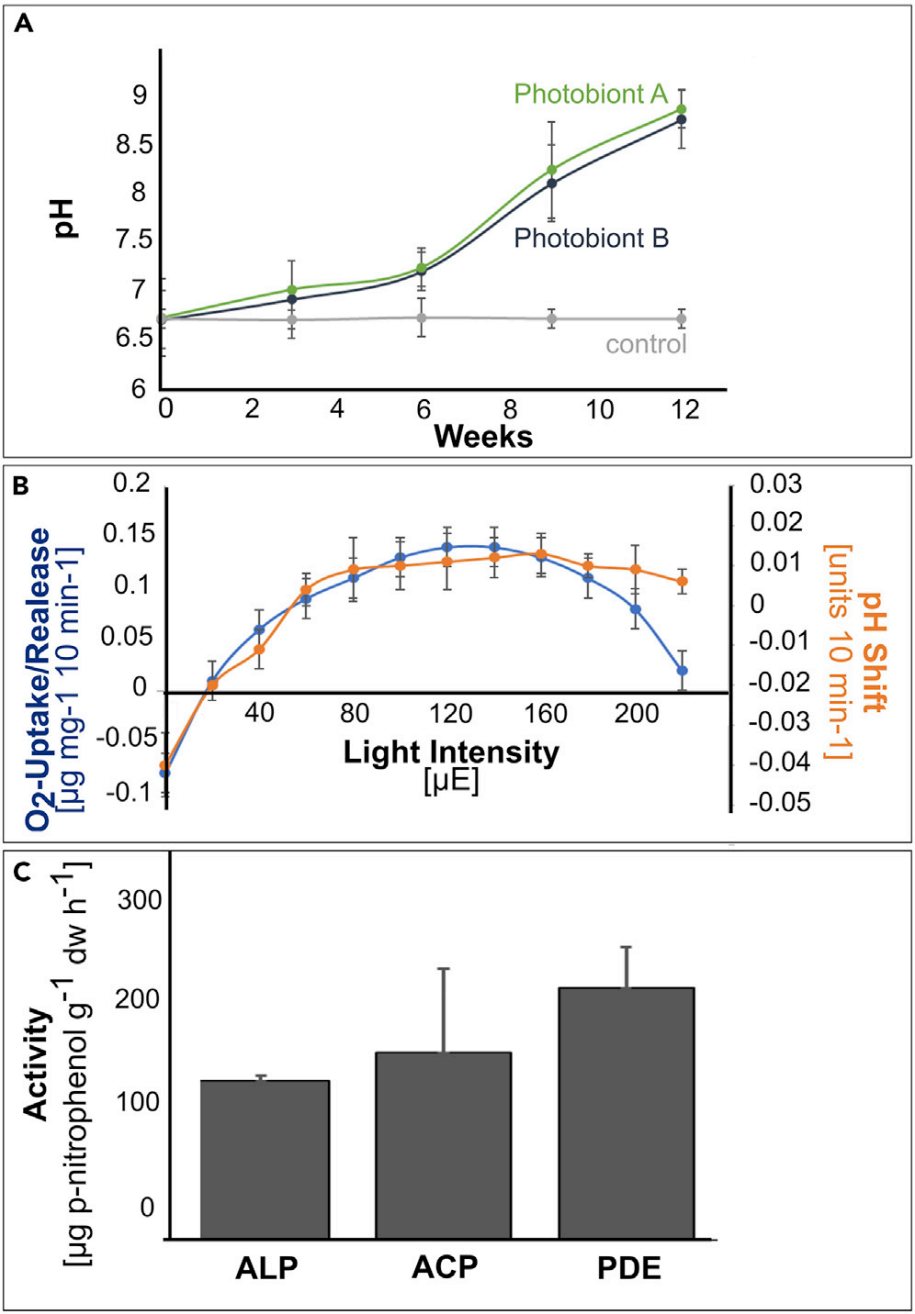
Alkalinolysis and Enzyme Activity of Grit Organisms (A) Long-term incubation of two isolated green algal photobionts (n = 3, for each photobiont and the control) indicating a raise in pH over the course of 12 weeks; (B) Short-term experiment with isolated green algal photobionts (n = 6) showing pH shifts depending on oxygen production during varying light conditions; (C) Enzyme activity of alkaline phosphatases (ALP), acidic phosphatases (ACP), and phosphodiesterases (PDE) (n = 3). Data represent mean +/− standard deviation.

During long-term pH measurements of photobiont culture media, a continuous rise in pH from 6.7 up to almost 9 was observed within 12 weeks (Figure 7A). Measurements with the oxygen electrode revealed that all samples containing algal suspensions isolated from single grit crust lichens released oxygen into the medium, and this process was accompanied by increasing alkalization with increasing light levels (Figure 7B). Below a certain light intensity (the so-called compensation point, when photosynthetic oxygen production compensates respiratory oxygen consumption), all samples started to acidify the solution. For all samples, a clear correlation between oxygen uptake/release and the pH was given. All samples alkalized the medium already at relatively low light intensities (<40 μE). Light levels beyond 150 μE caused a saturation in oxygen production. Black grit stones showed mean enzyme activities of 140, 165, and 221 μg p-nitrophenol g^−1^ dw h^−1^ for alkaline phosphatase, acid phosphatase, and phosphodiesterase, respectively (Figure 7C).

## Discussion

**Figure 8:**
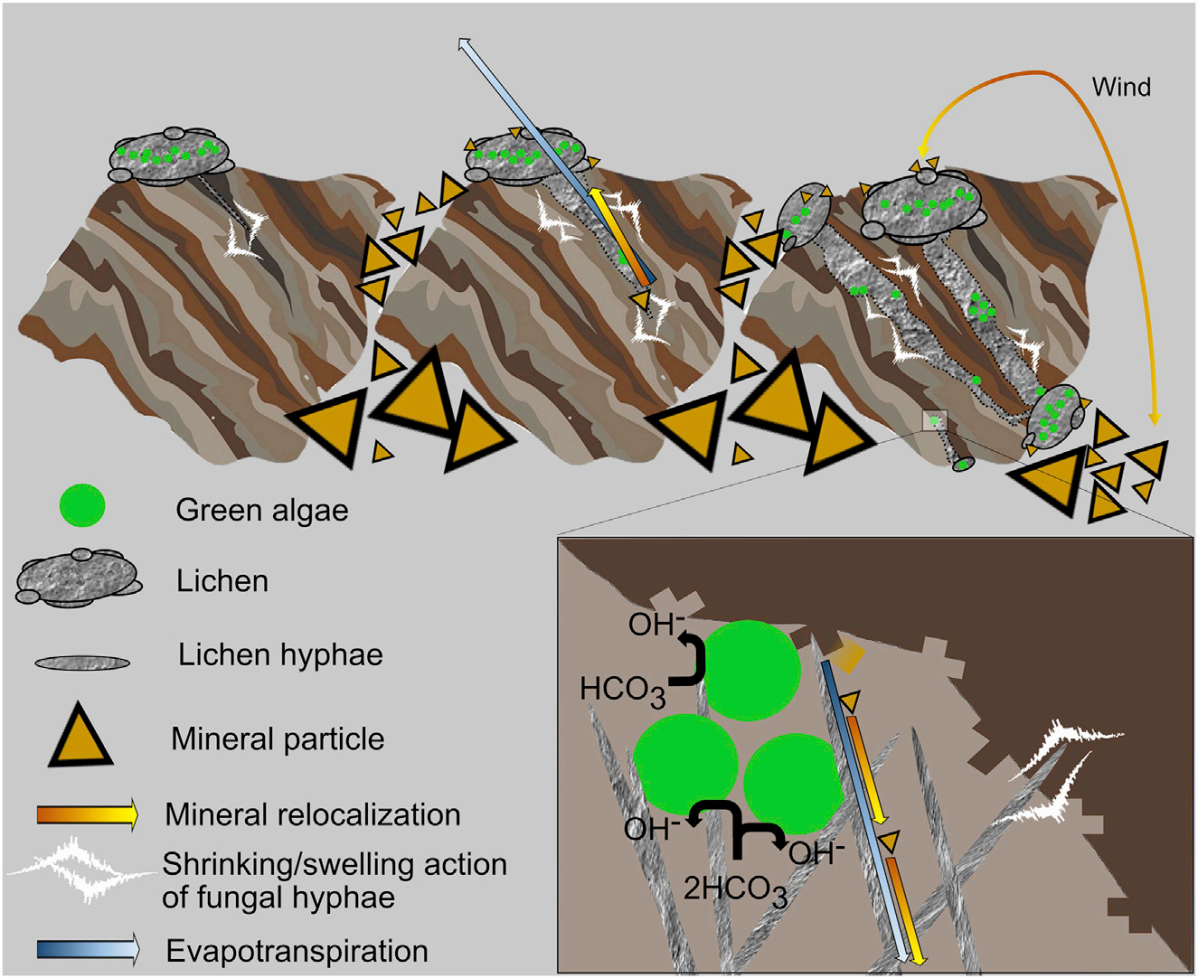
Schematic Overview of Bioweathering Processes Lichens consisting of a mycobiont and its hyphae (gray) together with green algal photobionts (green) colonize polycrystalline grit stones (brown tones) that have a size of roughly 6 mm. The hyphae of the lichen penetrate the lithomatrix causing tunnels induced by shrinking and swelling actions (white arrow) induced by fog and water input that hydrate the lichen. Further bioweathering activity (depicted in the inlet) leads to solubilization of minerals during times of hydration as a consequence of photosynthetic activity of the green algae photobionts. These excrete OH^−^ as a byproduct into the lithomatrix which causes alkalization that can promote the dissolution of e.g. quartz and other minerals. These minerals can be relocated (yellow arrow) following the direction of evapotranspiration (blue arrow) and re-crystallize (yellow triangles) on the surface of the lichen. On the surface of the lichens which is sticky due to excreted mucus like substances, mineral particles can accumulate between the grit stones from where they can be re-transported by wind (yellow arrow) which in turn can also lead to the accumulation of fine material on the lichen's surface.

The availability of water provided by fog, dew, and high air humidity in the region of Pan de Azúcar National Park in the Atacama Desert together with the presence of the grits as a substrate for colonization promotes the establishment of the grit crust as a landscape dominating aspect. As a consequence, this unique constellation of microbial life is in turn able to mediate bioweathering processes leading to physicochemical effects on the transformation of rocks. Considering the low amount of available water and relatively low chemical weathering susceptibility of quartz and feldspars in the granitoid rock substrate, biological and physical weathering appear to be the dominating processes during the very initial formation of soil. The large diurnal temperature range, for example (e.g. Jung et al., 2019a), causes thermal stress (insolation weathering) in the granitoid particles as differences in mineral expansion set up cause the polycrystalline rocks to crack preferentially at grain boundaries (Vasile and Vespremeanu-Stroe, 2017) which offers a great ecological niche for microbes of any kind. However, the bioweathering processes on which this study focused were those mediated by lichens, cyanobacteria and fungi that support the deterioration of rocks from a microscope to the landscape perspective and from the rock to the fine substrate as depicted in a possible chronosequence in Figure 1. However, the investigated and described processes are just observed in nature, as a result at the time of the sampling, not by following the course of development. Below several bioweathering processes such as enzyme activity, pH alterations, the incorporation of mineral fragments, shrinking-swelling activity of whole colonized grits and the re-localization of mineral fragments will be discussed on basis of the scheme summarized in Figure 8.

### Pore Spaces in Grit Particles

A common observation in thin sections of black grit samples was the presence of large pore systems (Figures 3D, 4A, and 4B), which were connected and open to the external surface (Jung et al., 2020), thus presenting a potential prerequisite for e.g. internal weathering reactions (Dultz et al., 2006). These pore systems may be the result of two different processes: (1) The pore system could at least in parts represent relicts of previous fluid-rock interactions, which took place e.g. in the hydrothermal phase after formation of the bedrock plutonite from which the granitoid grit particles originated. For the formation of cracks, fracturing during cooling of the plutonite, pressure relief by erosion and insolation weathering has to be considered (Wiggering, 1987). During dissolution of the minerals, a marked share of the parent material could have been lost to the liquid phase, hereby increasing the porosity (Putnis, 2002). The formation and precipitation of secondary phases (clay minerals and sesquioxides) may have counteracted this effect, in particular if elements were supplied from the surrounding solution. The presence of clay minerals in the pore system could have slowed down chemical weathering reactions as the surface of primary minerals could have been covered and pore diameters could have been narrowed by clay-sized particles. (2) Existing cracks can enable large diameter dissolution reaction by microbial colonization and relatively fast convective transport (Dultz et al., 2013) that may also have contributed to the observed pore system of the grit stones.

### Penetration of the Lithomatrix by Lichens

In Pan de Azúcar, lichen thalli established at the surface of granitoid substrate particles (Figure 4A), probably used the enhanced water condensation properties of the quartz containing substrate in a similar way as it was described for cyanobacteria by Azúa-Bustos et al. (2011). Once established, it can be speculated that the lichen started to alter the lithomatrix by drilling into the grit (Figure 8, gray hyphae inside grit) causing tramlines (superficial grooves) and channel formation on the surface, as well as cuts, holes, and borings (Figure 4D). These processes have been described in many other studies (e.g. Adamo and Violante, 2000). Often a significant pH reduction in the vicinity of cells upon mineral surface attachment or a significant turgor pressure of around 10–20 MPa that is applied toward the lithomatrix can be observed (Howard et al., 1991). This can lead to mineral mass loss at the interface of lithomatrices causing pits and tramlines. Those mass losses were estimated to account for ∼40%–50% of the overall bioweathering (Li et al., 2016). Additionally, the excretion of siderophores is also a common bioweathering strategy for microbes to overcome Fe deficiency (due to the strong tendency of Fe^3+^ to precipitate at circum-neutral pH). Siderophores chelate Fe-ions which can also lead to the dissolution of the lithomatrix (Neilands, 1995; Li et al., 2016). Near surface structures were found such as the brownish appearing material which was assigned to vermiculite or smectite clay minerals in combination with Fe-oxyhydroxides. These were interpreted as neoformations and are thus stable in longer terms due to the high insolubility of Al-dominated and Fe-dominated minerals at neutral pH (Banfield et al., 1999). Similar drilling processes and traces have been observed for a variety of fungi growing on e.g. limestone (Burford et al., 2003a, 2003b), but it remains unanswered if the grit crust organisms are actively causing the cracks or if they start growing at preexisting fissures. Evidence of a siderophore related bioweathering can be found in a significant enrichment of Fe in the second cm of the grit crust (Figure 3C) what might be the consequence of accumulated fine material.

The observed tunnels were not only colonized by fungi but also by phototrophs reaching deep inside of grits (Figures 4D and 8 gray hyphae and green photobionts inside the grits). Interestingly they were able to maintain their photosynthetic activity inside the grits due to the translucent character of the grits. With time, the lichen thalli then could invade the stone, which can be observed as embedded thalli (Figure 4C). This can lead to a direct contact even between the green algal photobionts in inner parts of the lichen thalli and the lithomatrix promoting additional types of bioweathring processes (Figure 8, inlet).

### Alkalinolysis, Acidification and Enzyme Activity of Grit Crust Communities

The pH-value of the white grit was higher (8.0) compared with that of black (i.e. = densely colonized) grit (6.9) (Table 1) indicating that the microorganisms might induce chemical weathering through the release of protons and organic acids.

Interestingly, the complementary experiments with the isolated photobionts of the lichens showed that trebouxioid green algae could increase the pH of a culture medium in the short-term, as well as in the long-term and that this was related to their photosynthetic activity (Figures 7A and 7B). During photosynthesis, green algae excrete OH^−^ as a byproduct, which in turn alkalizes the medium, a process known as alkalinolysis (Figure 8, inlet). This appears as a contradiction to the lower total pH of the black grit compared to the white grit but one needs to take into consideration that experiments with the isolated photobionts need to be interpolated to the *in situ* situation with care: for these experiments a high amount of biomass was required that cannot be found in nature to a comparable extend. Nevertheless, the process of alkalinolysis can be transferred to lichens because the photobionts were able to increase the pH in the experiments which means it is likely that they could do this in the lichens as well even during short terms of activity induced by e.g. fog (Jung et al., 2019a). This will result in alkalinolysis effects on the microscale where, e.g., a quartz fragment is stuck in the lichen thallus touching a few photobionts, which was indeed observed (Figure 5D). This will not cause a high pH in a bulk sample of several grams but a high pH at a certain spot within the lichen thallus, close to such a mineral fragment. These alkalinolysis processes have been described mainly for trebouxioid green algae (Shiraiwa et al., 1993; Weber et al., 2011) and some cyanobacteria such as *Chroococcidiopsis* (Büdel et al., 2004). These genera were found among the grit crust community (Jung et al., 2020). Interestingly, quartz as the main compound of the grit dissolves at pH > 7.5 (Brundsen, 1979) because here the solubility of mainly Si strongly increases, what could happen during alkalinolysis in the interface between photobionts and the lithomatrix of the grits.

In contrast to alkalinolysis, Salvadori and Municchia (2016) reviewed several acidic metabolites excreted by lichens and fungi as main weathering agents but also stated that this is depending on the lichen species and the climate. Although we did not detect any acidification reactions in our experiment with green algae, it is still possible that the fungal part of the lichen, the multicolonial fungi or other heterotrophic microorganisms can excrete acids or lichen compounds to deteriorate specific minerals within the grit stones.

Interestingly, enzyme assays revealed a comparably high activity of alkaline, acidic and diester-phosphatases (Figure 7C), which indicates that both processes, alkalization as well as acidification may have happened simultaneously but at different micro-sites. This may be explained by (i) the polycrystalline lithomatrix of the grit stones causing a scattered pattern of minerals with different probabilities of pH-shifts into the one or the other direction and (ii) the diverse taxonomic composition of the grit crust community which is not yet fully characterized.

### Wet-Dry Cycles

The arid component of the National Park is interrupted by fog and dew providing water for organisms (Lehnert et al., 2018). This constantly causes several wet-dry cycles per day (Jung et al., 2020). Poikilohydric organisms, such as lichens, cyanobacteria green algae and other heterotrophic microorganisms are able to use even these short periods of water availability e.g. with the help of excreted extracellular polymeric substances (EPSs). Swelling and shrinking movements of the organisms (Figure 8, whitish symbols) can cause or at least reinforce mechanical disruption of the lithomatrix. Our observations showed that a single grit stone colonized by lichens increased its volume by 21% upon wetting compared to air-dry conditions, with the lichen tissue alone increasing its volume by 55% (Figures 5A and 5B and Table 3). This demonstrates the high fragmentation potential of the microhabitat caused by the organisms in dependency of water input. Pure lichen thalli were reported to have water holding capacities up to 300% of their dry weight (Creveld, 1981), highlighting the deteriorating potential of lichens within such a system. The analysis of the surface area revealed that its increase occurred mainly at the surface of the grit (that is, of the lichen), while the (internal) interface of lichen and stone increased by less than 1%. One possible explanation for this pattern could be that the biomass of the organisms was probably much higher at the surface of the grits than in the internal structures. This was supported by fluorescence microscopy of grit cross sections showing a loose fungal network together with only a few green algae (Figure 4D). Here, the forces applied by shrinking and swelling were probably much weaker, but as the fog and dew events are cyclic on a regular basis a deterioration over time is likely. Meteorological records of the sampling sites for example showed that dew occurred frequently, predominately during night-time providing between 0.025 and 0.088 mm of liquid water per day but fog usually occurred during daytime providing higher water fluxes delivering 0.38–1.25 mm per day (Jung et al., 2020). However, it should be stressed that the lichens in our laboratory study were completely submerged in water for 10 min and then were given time to equilibrate overnight (12 h). It is possible that the behavior of the lichens would be somewhat different if they were subjected to the realistic environmental conditions (with water droplet supply from the gaseous phase) that they usually experience in the field.

In order to get a better understanding of the actual processes that are occurring in the field, a more sophisticated experimental design will be necessary in future studies. For example, the simulation of a changing water availability during dew or fog events would be possible with an environmental chamber such as the one described by Raanan et al. (2016). This would allow the quantification of the grit crust's structural dynamics during and at the end of fog or dew events in the Atacama Desert. For example, biological disruption of grits can be enhanced by the crystallization of salts within pores and cracks within the lithomatrix (Wellman and Wilson, 1965). Here analysis of the aqueous extract revealed distinct amounts of soluble salts in the samples (Table 1). In addition we observed Ca-containing phases accumulated next to the lichen thalli (Figure 5C), whereby the Ca might originate from dissolution of minerals. Inside the grit stones, Ca-containing P compounds may have been solubilized in water, ions been transported along the fungal network before mineral re-crystallization within the lichen thalli or the lichen's cortex as a consequence of evapotranspiration (Figure 8, brownish and blueish arrow) (Banfield et al., 1999). Further, this Ca-enrichment also could be a consequence of carbonatization performed by cyanobacteria or green algae.

### Mineral Accumulation

Besides Ca-containing compounds on top of the lichen thalli, mineral fragments were also found to be incorporated into the lichen thalli (Figure 4B). This was already described as mechanical action of crustaceous lichens on shale, shist, gneiss, limestone and obsidian by Fry, 1927. Barker and Banfield (1996) found that mineral fragments as small as 5 μm were incorporated into the lower thallus of lichens as a consequence of bioweathering processes on amphibole syenite. Further, we observed airborne dust particles which were embedded into the cortex of the thalli and which were concatenated by the fungal hyphae and EPS (Figures 6A, 6B, and 8, triangles on lichen's surface). Recently, it could be shown that up to 4 g m^−2^ of dust can accumulate at comparable sites in the Atacama Desert already within one month (Azua-Bustos et al., 2019). Comparable dust inputs have been shown to be trapped by cryptogamic ground covers of the Colorado Plateau enriching the soils with P, Mg, Na, K, Mo, and Ca thus highlighting the role of these surface consortia in fueling nutrient cycles (Reynolds et al., 2001). Dust at the Pan de Azúcar sampling site mainly consisted of Fe and Al containing compounds (Figure 6C) suggesting that these compounds probably originated from weathering products that were transported via dust over more or less long distances and thus could also be due to mining activities in the vicinity.

Nutrient elements such as P were part of the dust particles and co-occurred with Ca (Figure 6B) implying the presence of apatite (Ca_5_(PO_4_)_3_(F,Cl,OH)). Different lichen species interacting with apatite that carries P, one of the most growth-limiting elements in arid ecosystems (Zhao and Zeng, 2005), have recently been reported by Baumann et al. (2018) from another experimental site that was close to the sampling site of the present study.

### Pedogenesis and Accumulation of Available P

In the field, we observed that colonized grit stones were located on top of finer substrate (Figure 3A). Texture analysis revealed that almost 70 weight % in the second cm were substrate particles <2 mm (Figure 3B). We assume that this is a consequence of grit weathering during which parts of the lithomatrix broke off in the first cm and deposited as second centimeter (Figure 8, triangles). Further, deposition of secondary minerals may have contributed, as lichens were shown to be especially effective in altering feldspars to clay minerals or clay-sized particles even if this was the result of a long-term effect (Jackson, 2015). This is supported by significantly higher elemental concentrations of almost all investigated elements underneath the grits (Figure 3C). The enrichment in elements was also found for P, mainly occurring as relatively stable P (Figure 3D), which may include e.g. apatite- and phythate-P, as well as P occluded within sesquioxides (Baumann et al., 2018). These P-sources meet special demands of microorganisms as P supply is known to be one of the limiting factors for biological growth in arid ecosystems (Zhao and Zeng, 2005).

However, total C, C_org_ and N were only tendentially higher in the first compared with the second centimeter of the profile although the biomass of at least the phototrophic organisms was visibly concentrated in the first centimeter. In general, N accumulates in phototrophic communities because of the N fixing abilities of heterocytous cyanobacteria (Elbert et al., 2012), which were also found among the grit crust community (Jung et al., 2019b, 2020).

Also, the C_org_ concentration as a biomass proxy indicated similar biomass for both centimeters, which could be due to a vivid phototrophic community in the first centimeter and a well-established community of mainly heterotrophs in the second centimeter. In contrast, C_inorg_ was significantly higher in the second centimeter, which is in line with higher concentrations of Ca-containing compounds. They may have originated from grit stone weathering and/or dust deposition. Total C concentration, comprising C_org_ and C_inorg_ was highly variable and thus similar between the first and second centimeter. In addition, wind erosion in the Atacama Desert (McKay et al., 2003), leading to substrate input and output, may have affected elemental concentrations in both centimeters. However, the enrichment of fine particles appears to be the sum of (among others) bioweathering actions such as alkalinolysis of the lichen photobionts, drilling of the lichen's hyphae, dust trapping of biopolymers such as EPS and shrinking/swelling actions of microorganisms in internal structures of the lithomatrix. The bioweathering of rock material over time led to the formation of a *terrestrial protopedon* (Figure 8, triangles on the right side) (Jung et al., 2020), a young soil named in analogy to similarly young soils in aquatic ecosystems that are rich in CaCO_3_ with high contents of sand, silt or clay but which are poor in organic C (Amelung et al., 2018).

### Landscape Scenario and Significance

Considering that the climate of the Atacama Desert has been stable for 150 million years (Hartley at al. 2005) together with the ancient origin of lichens and algae it can be speculated that all of the described and simultaneously occurring weathering processes are part of an ongoing dynamic that takes place over geological timescales. Following this, the deterioration of rocks leads to the formation of smaller rocks over the formation of grits and finally to the terrestrial protopedon as a chronosequence (Figure 1). This appears to be a significant geomorphological process in parts of the Atacama Desert fueled by a community of microorganisms such as those of the grit crust. In this sequence the colonized grits could be seen as a snapshot of the present bioweathering stage at least in the landscape of Pan de Azúcar or there where the grit crust will be detected in future. Although this study focused mainly on lichens, algae and fungi it should be stressed that they are by far not the only active microorganisms in this system. Even a single lichen nowadays needs to be understood as a self-sustaining ecosystem formed by the interaction of an exhabitant fungus and an extracellular arrangement of one or more photosynthetic partners and an indeterminate number of other microscopic organisms (Hawksworth and Grube, 2020). Nevertheless we tried to assign specific bioweathering processes to single types of organisms because there are always surprises: recently, the extreme thermoacidophile *Metallosphaera sedula*, a metallophilic archaeon, was found to thrive on meteorite minerals (Milojevic et al., 2019) and other examples are *Manganitrophus noduliformans* and *Ramlibacter lithotrophicus* that were the first organisms that helped to untangle the mechanisms behind chemolithoautotrophy via manganese oxidation (Yu and Leadbetter, 2020). The great and so far unstudied diversity of microorganisms that are part of the grit crust biocenosis in the Atacama Desert might therefore be an interesting source for further bioweathering studies.

### Limitations of the Study

The authors want to clearly state that this study aims to give an overview on various interactions between the lithomatrix and different microbes without quantitatively investigating single processes. Only a very high number of replicates for each of the single weathering processes could be upscaled to the landscape scale at which they are taking place and at which several follow-up studies should focus in future. One of these studies will focus on a method to detect the shrinking and swelling actions of the grits induced by fog and dew mimicking more natural conditions in contrast to the lab-based approach of this study.

The contribution of this study also ends at untangling the detailed community structure of the grit crust and therewith cannot account for bioweathering activities of e.g. heterotrophic bacteria. Further, the degree to which dust deposition, local physical weathering, or the mining activity in the area contribute to the finer substrate layer could not be resolved here.

### Resource Availability

#### Lead Contact

Requests for materials and communications with the journal should be addressed to Patrick Jung (patrick_jung90@web.de).

#### Materials Availability

This study did not generate new unique reagents.

#### Data and Code Availability

All datasets used for this study are accessible through the lead author Patrick Jung (patrick_jung90@web.de).

## Methods

All methods can be found in the accompanying Transparent Methods supplemental file.

## Supplemental Information

Document S1. Transparent Methods
