# Supplementary material for: Lichens Bite the Dust – A Bioweathering Scenario in the Atacama Desert
Source: iScience. 2020 Oct 7;23(11):101647. doi: 10.1016/j.isci.2020.101647 (PMC7578742; doi:10.1016/j.isci.2020.101647)
Supplement: Document S1. Transparent Methods [file mmc1.pdf]

iScience, Volume *23*

Supplemental Information

Lichens Bite the Dust – A Bioweathering Scenario in the Atacama Desert

Patrick Jung, Karen Baumann, Dina Emrich, Armin Springer, Vincent J.M.N.L. Felde, Stefan Dultz, Christel Baum, Marcus Frank, Burkhard Büdel, and Peter Leinweber

Supplemental Information

Lichens bite the Dust – A Bio-weathering Scenario in the Atacama Desert

Patrick Jung, Karen Baumann, Dina Emrich, Armin Springer, Vincent J. M. N. L. Felde, Stefan Dultz, Christel Baum, Marcus Frank, Burkhard Büdel, Peter Leinweber

Supplemental Information

Transparent Methods

Site description

Pan de Azúcar National Park is located between 25°53′ to 26°15′S and 70°29′ to 70°40′W along the coast of Chile and is characterized by an arid climate (Lehnert et al., 2018; Baumann et al., 2018). Here, a narrow pediment close to the coast is followed by a steep mountain ridge reaching elevations up to 850 m a.s.l.. After this first mountain ridge, the terrain descends slightly to elevations between 400 and 700 m a.s.l. towards the inland. This study was conducted in 2.5 km distance from the Pacific coast (Lat: 25.96636111°S, Long: 70.61521111°W), where granitoid is the bedrock. The igneous rock granitoid is coarse grained and composed mostly of quartz, alkali-feldspar and plagioclase, whereas micas occur in minor amounts. Slightly altered granitoid rocks are exposed in the upper part of hills (Oeser et al., 2018; Bernhard et al., 2018), which have a conical shape due to weathering and erosion. On the bedrock as well as on rocks/stones of various sizes biocoenoses of lithobiontic communities were observed. On the grit-sized stones ('grus'), this biocoenosis even caused abundant blackish patterns in the landscape (Fig. 1) thus forming the so-called *grit crust* (Jung et al., 2020; called black grit). The heavily colonized blackish parts could be distinguished from only very little colonized whitish appearing parts (Jung et al., 2020; called white grit).

Sampling procedure

In July 2017, samples were collected from the grit-paved area. For petrographic descriptions, samples of black and white grit were collected. They were taken from three independent 25 x 25 cm plots each by removing the upper centimeter with a broad bristle brush. Similarly, samples for soil texture, element analyses and enzyme

activity analyses were collected from plots of black grit. For soil texture, elemental analyses, the same procedure was applied to the same plots to remove the second centimeter. Samples for geological thin sections, microscopy and X-ray computed microtomography analyses as well as photobiont isolation were taken by pressing five sterile Petri dishes into the ground (black grit). All samples were shipped and stored air-dry until further analyses.

Dust was collected from a plastic sheet (31 cm x 43 cm), which had been carefully attached to the ground by pegs. After 10 days of exposure to the open air, the dust was wiped off with a P-free filter paper, which was slightly wetted by double distilled water (ddH_2O) and placed in a Falcon tube for transport.

Mineralogical analyses of black and white grit samples

For mineralogical analyses, three field replicates of black and white grit (first cm), respectively, were combined. To release contained fine particles in grit stones the samples were finely ground in a ball mill to < 0.5 mm before the clay fraction < 2 µm was separated quantitatively by the Atterberg method by repeated sedimentation (11 times). Electrical conductivity (EC) and pH of the sample fractions were determined in a 1:10 aqueous extract (4.5 g sample and 45 mL deionized H_2O). The EC of deionized H_2O was 0.8 µS cm^{-1}. The concentrations of F^-, Cl^-, NO_2^-, Br^-, NO_3^-, PO_4^{3-}, SO_4^{2-}, Na^+, NH_4^+, K^+, Mg^{2+} and Ca^{2+} in the 1:10 aqueous extracts were quantified with a Metrohm IC instrument (930 Compact IC Flex). The standard solution with the lowest concentration was 0.25 mg L^{-1}. Measured values below this concentration were not evaluated. The validity of the determination of anions and cations was determined by calculating the charge balance ($mmol_c$) of anions and cations. Chemical composition of the two particle size fractions was determined with an XRF analyzer (S1 TITAN, Bruker; Middlesex, USA). The > 2 µm samples were gently ground with a mortar before

analysis of the dry powders. For identification of the clay minerals, the exchange sites of the < 2 µm fraction were saturated with Mg, transferred to glass slides and allowed to dry at room temperature. For the samples, X-ray diffraction patterns were obtained with an X-ray diffractometer (Siemens D500, Germany, Cu Kα radiation). The > 2 µm fractions were ground in a mortar and precipitated to glass slides before X-ray diffraction analyses.

Soil texture and element content of grit stones and dust samples

Soil texture was determined on a combined sample of three field replicates for black grit from the first and second cm, respectively, using sieving and sedimentation procedures after Blume et al. (2010). For elemental analyses, substrates of the first and second cm of three samples were ground to < 0.5 mm using a mixer mill (Retsch MM 200, Haan, Germany) operated at 30 Hz for 5 min with metal beads. Each sample was analyzed in duplicate. Total C, N, and S content was obtained by dry combustion using an elemental analyzer (VARIO EL, Elementar Analysensysteme GmbH, Hanau, Germany). Inorganic C (C_{inorg}) content was determined by Scheibler calcimeter and organic C (C_{org}) content was calculated by subtracting C_{inorg} from total C content (Blume et al., 2011).

For total elemental analysis, 0.5 g dry substrate or the whole filter paper with adhering dust (dust sample), respectively, was extracted using microwave-assisted digestion with aqua regia solution (3:1 hydrochloric acid : nitric acid) (Chen and Ma, 2001; ISO standard 11466). The elemental concentrations were then determined by inductively coupled plasma optical emission spectroscopy (ICP OES) (JY 238 UL Trace, France). Sequential P fractionation was carried out after Hedley et al. (1982), with slight modifications. Solubility of the fractions was assigned to labile P (resin-P and $NaHCO_3$-P), moderately labile P (NaOH-P), relatively stable P (H_2SO_4-P) and non-extractable P

(residual-P). Total P concentration in the fractionation extracts was determined by ICP OES at 214 nm wavelength.

Scanning electron microscope – energy dispersive X-ray analysis (SEM-EDX)

To visualize structures and elemental distributions on single black grit stones, a field emission scanning electron microscope (SEM, MERLIN® VP Compact, Carl Zeiss Microscopy GmbH, Oberkochen, Germany) equipped with an energy dispersive X-ray (EDX) detector (XFlash6/30, Bruker Nano GmbH, Berlin, Germany) was used. Before analysis, the grit-stones were fixed by hot glue on 0.5"SEM Pin Stubs (agar scientific; Plano GmbH, Wetzlar, Germany) and coated with carbon under vacuum (EM SCD 500, Leica Microsystems GmbH, Wetzlar Germany). SEM images were taken at 5kV up to 25 kV and elemental distributions were analyzed using SEM-EDX Quantax Esprit software (version 2.0, Bruker Nano GmbH, Berlin, Germany).

Preparation and microscopy of thin sections and cross sections of lichen thalli

To visualize colonization of the grit stones, thin sections of single black grit stones were prepared. Grit stones including inhabiting organisms (n = 50) were fixed, dehydrated and embedded in acrylic resin (LR-white; London Resin Company, London, UK) according to Bungartz et al. (2004). All steps were conducted in a vacuum chamber. The embedded samples were polished on one side, fixed to glass slides and polished to a thickness of approximately 30 µm. In addition to light microscopy the thin sections were investigated under a fluorescence microscope (Axioskop; HBO 50; Carl Zeiss, Jena, Germany) with 200-fold magnification and AxioVision software (Carl Zeiss, Jena, Germany). The autofluorescence of the green algal photobionts (red) as well as of the fungal hyphae of the lichens (white) was visualized (blue-violet 395-440 nm excitatory 577 filter; 460 nm chromatic beam).

In addition to the thin sections, also cross sections of hydrated lichen thalli were prepared by using lichen thalli, which were detached from black grit stones. They were cut with a cryo-microtome to visualize the inner structure of the thalli. The 20 to 25 µm thin cross sections were transferred with a fine brush to a drop of water on an objective slide, covered with a cover slip and investigated by light microscopy (Axioskop, Carl Zeiss, Jena, Germany) using oil immersion and a 630-fold magnification.

X-ray computed tomography analysis

To investigate the swelling and shrinking potential of single black grit stones during wet-dry cycles, computed microtomography (µCT) scans were done using a Zeiss Xradia 520 versa (Jena, Germany). A single colonized grit stone was first scanned in a dry state, using 80 keV and 1600 projections with an acquisition time of 3 sec. The resulting voxel edge length was 13.41 µm. The projections were then reconstructed as an 8 bit greyscale tiff image stack with 1012 slices. After the first scan, the sample was submerged in tap water for 10 min, to allow the lichen tissue to become fully hydrated. Then, the grit stone was placed in a closed falcon tube and allowed to equilibrate in the dark overnight, in order to prevent the sample from swelling or shrinking during the second scanning process, as this would have resulted in blurred images. The same scanning parameters were used for the second scan of the hydrated grit stone.

Image analysis was done using the software ImageJ (Schindelin et al., 2012). For noise reduction, we used the non-local means denoising algorithm (Buades et al., 2011) with default settings. This filter works like Gaussian blurring, but preserves edges and boundaries. The segmentation was done using the trainable WEKA segmentation plugin for ImageJ (Arganda-Carreras et al., 2017). This plugin is very effective for multiphase segmentation because it combines a collection of machine learning algorithms with a set of selected image features to produce pixel-based segmentation.

Briefly, this plugin can be trained to learn from user input on a training dataset to later perform the same task on an unknown dataset. After manually selecting stone, lichen and pore space, the plugin needed three training runs until it produced a very precise classification of the three phases.

After the segmentation, a median filter with a window size of 2 voxel was used to remove individual isolated voxel from the segmented image. The volume of the different phases was measured by counting the voxels and multiplying them with the voxel resolution. The surface was measured using the “isosurface” algorithm of the BoneJ plugin in ImageJ (Doube et al., 2010). This feature uses marching cubes to create a triangular surface mesh and calculates the object surface as the sum of the areas of the triangles (Lorensen and Cline, 1987). For the visualization of the scans, we used the Vaa3d Software Version 3.2 (Peng et al. 2014), as well as the volume viewer plugin from ImageJ.

Photobiont isolation and culturing

Two single lichen thalli from black grit stones of the first cm were picked manually and crushed in 100 µL ddH_2O before plated on petri dishes with Bold’s Basal Medium (BBM; Bischoff and Bold, 1963) with 1.5 % agarose under sterile conditions. After four weeks two photobiont colonies were picked with a sterile needle under a binocular microscope and plated on fresh BBM plates with 1.5 % agarose. After additional four weeks the plates were checked for pureness and the two pure photobiont colonies were transferred to 200 mL liquid BBM medium as photobiont stocks. Both stock cultures were maintained at 20 µE m^{-2} s^{-1} and a light–dark regime of 16:8 hours at 17 °C. The cultures were shaken several times per day to allow gas exchange with the atmosphere for four months.

Long term pH development of photobionts

To detect the pH development of lichen photobionts, 10 mL of each of the two photobiont stocks were added to 190 mL of liquid BBM media under sterile conditions. Three replicates for each lichen photobiont were prepared in this way and cultivated under the conditions described together with three replicates of 200 mL sterile BBM medium as controls. Every three weeks, 15 mL of each culture and of the controls were decanted into Falcon tubes and the pH was measured with a pH electrode (Meter Lab PHM210, ± 0.01; Radiometer Analytical SAS; Lyon, France). Each replicate was measured three times and a mean value was calculated. The measurements were done in week 0 (starting point of the experiment) and then repeated every three weeks up to 12 weeks.

Short term O_2 and pH development of photobionts

To monitor short term O_2 and pH development of photobionts, experiments were prepared adding 10 mL of each photobiont stock solution to 90 mL of liquid BBM. Three replicates for each photobiont were prepared in this way in glass bottles. During the experiments, which followed Weber et al. (2011), the suspensions were stirred by a magnetic mixer to ensure a gas exchange with the atmosphere in the culture room at 17 °C. Each sample was kept in the dark for 30 minutes for adaptation and pH and O_2 was measured every 2 minutes during an interval of 10 additional minutes in the dark. Oxygen was measured with an O_2 electrode (WTW FDO 925, 0.00-20.00 mg L^{-1} ± 0.5% v. Mw.; Xylem analytics GmbH and Co KG, Weilheim, Germany) and pH with a pH electrode (Meter Lab PHM210; Radiometer Analytical SAS; Lyon, France), which were fixed to the glass bottle to have continuous measurements of both, oxygen and pH. Afterwards the suspensions were exposed to an LED-light panel (PG8 14W LED

Plant Grow Light, Excelvan) as light source to measure pH and O_2 development at different light levels (20, 40, 80, 120, 150, 200, 220 µE). The different light levels were achieved by altering the distance between the light panel and the samples which was controlled with a universal light meter (ULM-500, WALZ, Effeltrich, Germany) that was mounted to the glass bottle. The algal suspensions were allowed to adapt for 10 minutes during exposure of the first light level (20 µE) and pH and O_2 was measured at this light intensity every 2 minutes during additional 10 minutes. Afterwards the samples were exposed to the next light level with a one-minute adaptation period. After the experiments, 1 mL of each algal suspension was taken out and centrifuged. The pellet was re-suspended in 4 mL DMSO and the $chlorophyll_{a+b}$ content was extracted and calculated following Ronen and Galun (1984) as a proxy for the biomass of each culture.

Determination of enzyme activity

From three replicates of each black grit sample (first cm, unground), alkaline phosphatase (ALP), acid phosphatase (ACP), and phosphodiesterase (PDE) activity were determined after Tabatabai and Bremner (1969) and Eivazi and Tabatabai (1977). The enzyme activity was measured in µg p-nitrophenol discharged from a pre-given p-nitrophenylphosphate solution in 1 g material within 1h (µg p-nitrophenol $(g\ h)^{-1}$).

Statistics

The Shapiro Wilk and Levene's test were used to test the elemental parameters of the first and second cm substrate for normal distribution and homogeneity of variances, respectively. A paired t-test was applied to reveal significant differences between the elemental concentrations of the first and second centimeter. All statistical analyses

were performed using R software version 3.5.3 (R Core team, 2019). Unless otherwise noted, significant differences refer to $P \leq 0.05$.
